# MARITAL STATUS TRANSITIONS AND INFLAMMATION DURING MID AND LATER LIFE: EVIDENCE FROM THE HEALTH AND RETIREMENT STUDY

**DOI:** 10.1093/geroni/igad104.1414

**Published:** 2023-12-21

**Authors:** Jeffrey E Stokes, Heather Farmer, Sarah Patterson

**Affiliations:** University of Massachusetts Boston, Boston, Massachusetts, United States; University of Delaware, Newark, Delaware, United States; University of Michigan, Ann Arbor, Michigan, United States

## Abstract

Research has increasingly utilized inflammatory markers to understand how social factors influence health, yet little research to date has examined marital status, or–crucially–later-life changes to marital status in relation to inflammation. Further, few studies have examined variation in such associations by gender, race/ethnicity, or age. We used data from the 2016 Health and Retirement Study (HRS) Venous Blood Study (n=9,933), which captures five inflammatory markers: C-reactive protein (CRP), Interleukin-6 (IL-6), Interleukin IL-10 (IL-10), Interleukin-1 receptor antagonist (IL-1Ra), and tumor necrosis factor alpha (TNF). Information concerning marital status came from the 2014 and 2016 waves of HRS, to ascertain stability of and changes to marital status over 2 years. Interactions were examined between marital status/transition groups and participants’ gender, age, and race/ethnicity. Overall, results were consistent across outcomes. Across 4 of 5 outcomes, the Newly Widowed and Never Married exhibited higher inflammation than the Continuously Married. Further, relationships between Continuously Widowed status and higher CRP and IL-6, and between Newly Widowed status and higher IL-1Ra, were present only for men. The association of Continuously Widowed status with higher CRP and IL-6 weakened with age, as did the effect of Newly Married status on IL-6. No clear patterns were identified concerning interactions with race/ethnicity. Findings indicate that marital transitions may have potential to harm physiological health. Negative consequences of widowhood for inflammation were also greater for men than for women, yet these harms may be attenuated at older ages when widowhood is an “on-time” rather than an “off-time” event.

